# Gait Characteristics of Children with Spastic Cerebral Palsy during Inclined Treadmill Walking under a Virtual Reality Environment

**DOI:** 10.1155/2019/8049156

**Published:** 2019-08-19

**Authors:** Ye Ma, Yali Liang, Xiaodong Kang, Ming Shao, Lilja Siemelink, Yanxin Zhang

**Affiliations:** ^1^The Research Academy of Grand Health, Faculty of Sport Science, Ningbo University, Ningbo, China; ^2^Bayi Rehabilitation Center, Chengdu, Sichuan, China; ^3^Motekforce Link, Netherlands; ^4^Department of Exercise Sciences, The University of Auckland, New Zealand

## Abstract

**Objective:**

To investigate gait characteristics in children with spastic cerebral palsy during inclined treadmill walking under a virtual reality environment.

**Methods:**

Ten *spastic cerebral palsy* (CP) children and ten *typically developing* (TD) children were asked to walk at their comfortable speed on a treadmill at a ground level and 10° inclined. Three-dimensional kinematic data and ground reaction force data were captured in a computer-assisted rehabilitation environment system. Kinetic parameters and dynamic balance parameters were calculated using a standard biomechanical approach.

**Results:**

During uphill walking, both groups decreased walking speed and stride length and increased peak pelvis tilt, ankle dorsiflexion, and hip flexion. Compared with TD children, CP children had decreased walking speed and stride length, decreased peak hip abduction moment, increased stance phase percentage, increased peak ankle dorsiflexion and knee flexion, and increased peak hip extension moment. The peak trunk rotation angle, ankle angle at initial contact, and stride length showed a significant group∗walking condition interaction effect.

**Conclusions:**

CP children showed similar adjustments for most gait parameters during uphill walking as TD children. With a lower walking speed, CP children could maintain similar dynamic balance as TD children. Uphill walking magnifies the existing abnormal gait patterns of the cerebral palsy children. We suggest that during a treadmill training with an inclination, the walking speed should be carefully controlled in the case of improving peak joint loading too much.

## 1. Introduction

*Cerebral palsy* (CP) is a neurological disorder that results from defects or damages of the immature brain [1, 2]. Problems caused by CP, such as muscle tightness, weakness, or spasticity, could impede musculoskeletal development and thus result in abnormal gait patterns [3].

Improving walking ability is one of the major concerns in therapeutic interventions for children with CP. Treadmill walking has been widely used in the rehabilitation of CP children to provide repetitive training of the whole gait cycle [4–7]. A systematic literature review evaluated the effectiveness of treadmill training for CP children [8]. The review suggested that treadmill training is a safe and feasible method for CP children and can improve walking speed and general gross motor skills. Willerslev-Olsen et al. [9] investigated the effect of inclined treadmill training on CP children. Their study suggests that inclined intensive gait training increases beta and gamma oscillatory drive to ankle dorsiflexor motor neurons and therefore improves toe lift and heel strike in CP children.

The biomechanical studies including kinematics, kinetics, and dynamic balance analysis are helpful for gaining insight into the neural control strategies, understanding the abnormal gait patterns thoroughly, and designing effective therapeutic interventions for CP patients. Kinematics is used to quantify the abnormalities of gait patterns [10–12]. Kinetics provides an indication of the causes of the gait abnormalities and the underlying muscle function pathology [10]. Healthy people can adapt to uphill walking by increasing hip, knee, and ankle dorsiflexion and thus maintaining an upright posture [13]. This adaptation can be used as a targeted training of a group of muscles (ankle dorsiflexor, knee extensor, and hip extensor). However, CP children might have difficulties in adjusting to inclined walking due to impaired postural control or dynamic balance.

Biomechanical studies are limited for inclined treadmill gait training on CP children. Several studies investigated the biomechanical characteristics and gait adaptation strategies of CP children for walking on an inclined ramp or treadmill [13–16]. These studies report that CP children adapt to inclined walking with similar gait adjustment strategies as the *typically developing* (TD) children but use greater postural adaptations.

To the best of our knowledge, there is a lack of thorough understanding of abnormal gait patterns for children with spastic CP during inclined treadmill walking using *three-dimensional* (3D) gait analysis including kinematics, kinetics, and dynamic balance analysis. Only kinematic data is reported in most of the aforementioned studies [14, 15]. The use of *two-dimensional* (2D) motion cameras [15] also loses considerable measurement accuracy for this data.

This study is aimed at comprehensively investigating gait adjustment strategies of CP children in level treadmill and uphill treadmill walking under a virtual reality environment (a default setting for a *computer-assisted rehabilitation environment* (CAREN) system; Motekforce Link, the Netherlands). The study quantified spatial-temporal parameters, 3D kinematics, 3D kinetics, and dynamic balance of the CP children by using the state-of-the-art motion capture techniques. We hypothesized that (1) CP children used similar gait adjustment strategies as their TD peers during inclined walking and (2) the CP group would have significantly lower postural stability due to impaired postural control.

## 2. Methods

### 2.1. Study Design and Subjects

Ten spastic CP children (age: 8.5 ± 2.3 years old; height: 131 ± 13 cm; weight: 28 ± 6.9 kg) and ten TD children (age: 7.9 ± 1.4 years old; height: 132.5 ± 11 cm; weight: 26.8 ± 6.3 kg) were included. The characteristics of the CP participants are presented in Table 1. There are no significant differences in age (*p* = 0.478), height (*p* = 0.494), or weight (*p* = 0.255) between the two groups.

The inclusion criteria for CP children are as follows: (1) diagnosed with diplegic CP, (2) 6-12 years old, (3) ranked I-II in the *Gross Motor Function Classification System* (GFMCS), (4) capable of understanding and executing instructions, (5) independent walkers without assistance for more than six minutes, and (6) with no botulinum toxin in the lower extremities or surgery during the preceding six months. The exclusion criteria for both CP and TD children are the absence of (1) severe heart and lung diseases and (2) visual or auditory system disorders. The ethical approval was obtained from the Sichuan Bayi Rehabilitation Center's ethics committee (Sichuan, China). Children's parents signed the consent forms for participation.

### 2.2. Instrumentation

**Figure 1:**
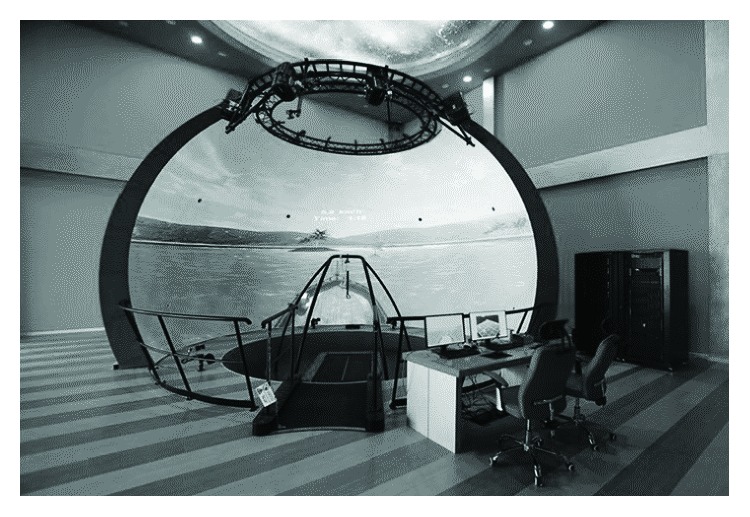
The CAREN system used for this study.

*Three-dimensional* (3D) joint kinematics and *ground reaction force* (GRF) were collected using a *computer-assisted rehabilitation environment* (CAREN) system. The CAREN system is an immersive virtual environment system consisting of a 3D motion capture system with twelve high-speed infrared cameras (Vicon, Oxford Metrics, UK), a split-belt force plate instrumented treadmill (ADAL3DM-F-COP-Mz, Tecmachine, France) atop a six degree-of-freedom motion base platform, and a cylindrical projection system. A safety harness and side rails are placed to ensure the safety and comfort of the user (see Figure 1). The Vicon motion capture system recorded kinematic data at a sampling frequency of 100 Hz. The force plate data were recorded with a sampling frequency of 1000 Hz. The visual scene is usually synchronized with the movement of the platform or the motion of the patient.

The CAREN system is employed in this study due to the following concerns: (1) the CAREN system can perform 3D movement for a full body in real time, which provides immediate feedback to both the therapist and patient [17]; (2) the CAREN system can conduct inclined walking experiment and collect kinematic and kinetic information simultaneously; (3) the virtual environment is reproducible and as close to a natural environment as possible [18, 19]; (4) the CAREN system is proved to be an effective tool for rehabilitation (such as gait training [17, 20], prosthetic adjustment [21], balance training [22, 23], and cognitive rehabilitation [24]) and biomechanics research [25–27].

### 2.3. Experimental Protocol

The motor functioning information (described by the GMFCS ranking) for CP and the classification of CP subtypes were obtained from each CP child's medical record. The participants were fully instructed before the measurements. Each participant started with a familiarization of three minutes on the treadmill at zero and a ten-degree inclined slope (uphill), respectively. The familiarization finished until the participant adapted to the walking conditions with a comfortable walking speed for each condition.

After changing clothes and shoes, 25 retroreflective markers were placed on the participant's anatomical landmarks following the definition of the *whole body human body model* (HBM) [26]. The markers are placed on the 10^th^ thoracic vertebra, navel, sternum, anterior superior iliac spine, posterior superior iliac spine, greater trochanter, lateral epicondyle of knee, lateral malleolus, posterior calcanei, the tip of big toe, lateral fifth metatarsal heads, acromion, lateral epicondyle and medial epicondyle of the elbow, lateral wrist, medial wrist, xiphoid process, the 7^th^ cervical vertebra, top of the head, right side of the head, and left side of the head.

Local segment coordinate systems were set up for the torso, pelvis, thigh, shank, and foot segments based on recorded markers' positions, which are listed in Table 2 (see more details from [26]).

For each sampling time frame, the coordinates of each segment with respect to its proximal segment were transformed by a sequence of three rotations delineated by three Euler angles following the flexion/extension, adduction/abduction, and internal/external order.

For safety considerations, the participants wore a harness which was fastened to a metal frame using a safety line throughout the experiment. Every participant was asked to perform a static trial to locate the positions of the anatomical landmarks and the locations of the joint centers. Then, each participant walked at their comfortable speed without handrail support in the virtual environment (a virtual walkway) projected on a cylindrical screen. The data were recorded for one minute during level treadmill walking. Subsequently, the platform was tilted at ten degrees uphill. Uphill walking data were recorded for one minute as well.

### 2.4. Data Processing

The study used a commercial software system, named the *human body model* (HBM) [26], embedded in the D-flow of the CAREN system [25], to calculate kinematics and kinetics. For the kinematic data and the GRF, the cutoff frequency of the low-pass filter was set to 6 Hz.

The HBM solves the inverse kinematics problem using a nonlinear least squares problem (1). The inverse dynamic solution is to find an optimal pose *Q* that best fits the maker data. In equation (1), $\vec{r_{i}}\left(Q\right)$ is the 3D position of a marker *i* and ${\vec{r}}_{i,\text{meas}}$ is the marker coordinates measured by the motion capture system.

(1) $$\begin{array}{c} Q=\arg\min_{Q}\overset{N}{\underset{i=1}{\sum }}{\left\|\vec{r_{i}}\left(Q\right)-{\vec{r}}_{i,\text{meas}}\right\|}^{2}. \end{array}$$

The HBM solves the inverse dynamic problem using the typical multibody equation of motion (2).

(2) $$\begin{array}{c} \tau =M\left(Q\right)\ddot{Q}+c\left(Q,\dot{Q}\right)+G+E, \end{array}$$

where *τ* is the unknown joint moments and forces, *M*(*Q*) is the human body mass matrix, *c* is the centrifugal and Coriolis loading, *G* is the gravity, and *E* represents the external force.

The *center of pressure* (COP) position was measured by the instrumented treadmill. The *center of mass* (COM) position was calculated based on measured kinematic data using a standard procedure as described by Winter, which determined the whole body COM based on the COM from individual body segment [28]. COP-COM separation in both the *anterior-posterior* (AP) and *medial-lateral* (ML) directions, the distance between COM and COP in the AP and ML directions, was calculated to represent the dynamic balance during gait [29]. To cater both the left footed and right footed trials, the COP-COM separation in the ML direction is made positive for all trails. These positive values reflect the distance of the feet which were being placed on either side of the COM in the ML direction. The average COP-COM separation in the AP and ML directions is normalized to each participant's leg length to allow for a comparison between subjects. Assuming that both legs have equal lengths, leg length was calculated as the distance between the left hip joint center and left ankle joint center during the static trial.

### 2.5. Statistical Analysis

Spatial-temporal, kinematic, kinetic data, and dynamic balance parameters were analyzed. Low reliability and large errors have been reported for the hip and knee transverse plane angles and knee frontal plane angles recorded by 3D motion capture systems [30]. These parameters were not included in this study.

Eight gait cycles from each participant under each walking condition were selected for the analysis. The Shapiro–Wilk test was performed to test the normality of the data. A two-way mixed-design analysis of variance (ANOVA) (group∗walking condition) was used to analyze the spatial-temporal, kinematic, and dynamic balance parameters using SPSS 22.0. For kinetic parameters (joint moments), a two-way ANCOVA (group∗walking condition) with speed as a covariate was used. A statistically significant difference was accepted as *p* < 0.05. The eta squared (*η*^2^) is used as the measure of the effect size. The *η*^2^ of 0.01, 0.06, and 0.14 means the small effect, moderate effect, and large effect, respectively [31].

## 3. Results

### 3.1. Spatial Temporal Parameters

As shown in Table 3, a significant difference is identified in walking speed between CP and TD children (*p* < 0.01, *η*^2^ = 0.749). Both groups decreased walking speed during uphill walking (*p* < 0.01, *η*^2^ = 0.737). The interaction effect of the walking speed (group∗walking condition) does not reach a statistical significance. The stride lengths of the CP children are shorter than those of the TD children (*p* < 0.01, *η*^2^ = 0.516). Both groups decreased stride length significantly during uphill walking (*p* < 0.01, *η*^2^ = 0.581). There is a significant difference in the interaction effect (*p* < 0.01, *η*^2^ = 0.388) of the stride length.

The CP children show a significantly longer stance phase compared to the TD children (*p* < 0.01, *η*^2^ = 0.523). Both groups increase stance percentage during uphill walking compared to level treadmill walking (*p* < 0.01, *η*^2^ = 0.557), with a significant group∗walking condition interaction effect (*p* < 0.01, *η*^2^ = 0.298).

### 3.2. Joint Kinematics and Dynamic Balance

As shown in Table 3, CP and TD children increase peak pelvic anterior tilt when walking uphill (*p* < 0.01, *η*^2^ = 0.842). CP and TD children have less peak pelvic posterior tilt (*p* < 0.01, *η*^2^ = 0.843), peak pelvis oblique (*p* < 0.01, *η*^2^ = 0.423), and less peak trunk extension (*p* = 0.026, *η*^2^ = 0.245) when walking uphill (*p* < 0.01, *η*^2^ = 0.843). Kinematic data shows significant differences for peak hip abduction during the swing phase (*p* = 0.026, *η*^2^ = 0.309), peak hip flexion (*p* < 0.01, *η*^2^ = 0.752) during the swing phase, and decreased peak hip extension during the stance phase (*p* < 0.01, *η*^2^ = 0.478) during uphill walking in both groups. Compared to level treadmill walking, uphill walking has a significantly smaller distance between COM and COP in the *anterior-posterior* (AP) direction (*p* < 0.01, *η*^2^ = 0.190).

CP children walk with a lower peak knee flexion angle during the swing phase than TD children (*p* < 0.01, *η*^2^ = 0.439). Both groups flex the knee more when walking uphill (*p* < 0.01, *η*^2^ = 0.539). There is a significant group∗walking condition interaction effect (*p* < 0.01, *η*^2^ = 0.238). At initial contact, CP has more knee flexion than TD (*p* < 0.01, *η*^2^ = 0.614). Both groups increase peak knee flexion during the load responding phase when walking uphill (*p* < 0.01, *η*^2^ = 0.825).

There is no significant group∗walking condition interaction effect in peak ankle dorsiflexion. Both groups increased peak ankle dorsiflexion during the stance phase when walking uphill (*p* < 0.01, *η*^2^ = 0.721). CP children show decreased peak plantarflexion compared to TD children during the swing phase (*p* < 0.01, *η*^2^ = 0.656). Both CP and TD decrease their peak plantar flexion during the stance phase and swing phase when walking uphill (*p* < 0.01, *η*^2^ = 0.598). CP has higher ankle dorsiflexion than TD at the initial contact. Significant differences of the ankle dorsiflexion at the initial contact are identified in the main effect for the group (*p* < 0.01, *η*^2^ = 0.362), walking condition (*p* < 0.01, *η*^2^ = 0.863), and the interaction effect (group∗walking condition) (*p* < 0.01, *η*^2^ = 0.357). The peak trunk rotation angle shows a significant group∗walking condition interaction effect (*p* = 0.017, *η*^2^ = 0.470).

### 3.3. Joint Kinetics

As shown in Table 3, both CP and TD children decrease hip peak flexion moment during the stance phase when walking uphill (*p* < 0.01, *η*^2^ = 0.817). CP children have greater peak hip extension moment than TD children (*p* < 0.01, *η*^2^ = 0.565) during the stance phase. The main effect for the walking condition also shows that peak hip extension moments during the stance phase increased when walking uphill (*p* < 0.01, *η*^2^ = 0.638). Peak knee flexion moment and extension moment during the stance phase do not show significantly main effects in the group and walking condition. CP children have lower peak ankle dorsiflexion moment in the stance phase than TD children (*p* < 0.01, *η*^2^ = 0.623). Lower peak ankle dorsiflexion moments in the stance phase are found both in CP and in TD children during uphill walking compared to level ground walking (*p* < 0.01, *η*^2^ = 0.416). CP children have reduced peak ankle plantarflexion moments in the stance phase compared to TD children (*p* < 0.05, *η*^2^ = 0.480). Significant between-group differences are observed for peak hip abduction moment in the stance phase (*p* = 0.018, *η*^2^ = 0.340).

## 4. Discussion

The study is aimed at investigating gait characteristics during inclined treadmill walking under a *computer-assisted rehabilitation environment* (CAREN) system in children with CP. The CAREN system, which is employed in our study, is appropriate for cognitive and physical rehabilitation training or assessment owing to its ability of creating realistic environments and collecting multisensory research data. Studies on postural control training in the CAREN system show that a single training session is enough to trigger an adaptation process of balance [32] and there is no significantly different COP displacement between the subjects who participate in the virtual environment and those who do not [33]. Walking characters including temporal-spatial parameters and kinematics in treadmill walking using CAREN system and over ground walking have no significant difference. Visual perturbations are not involved in our experiment design. Thus, the gait characteristics are comparable with other studies, which do not use a virtual environment.

Our results reveal that CP children had significant gait changes in several spatial-temporal, kinematics, and kinetics parameters when walking uphill. The changed gait characteristics include decreased walking speed and stride length and increased peak pelvis tilt, peak ankle dorsiflexion (during the stance phase), hip flexion, and knee flexion (during the stance phase). Decreased peak hip abduction in the swing phase and increased peak pelvis oblique angles are also observed. In general, CP children show similar gait adjustments as TD children during uphill walking.

**Figure 2:**
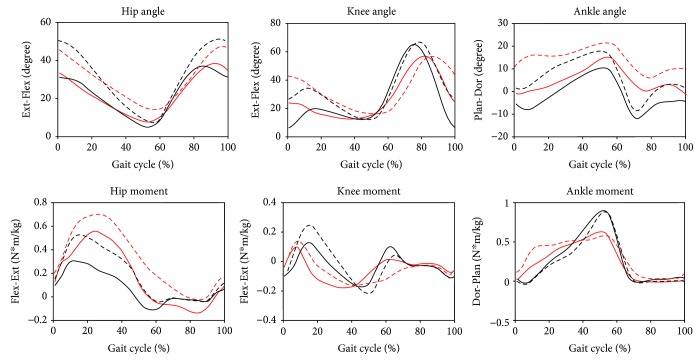
Mean joint angles and joint moments for CP and TD during level ground walking and uphill walking (solid black line: TD level walking; dashed black line: TD uphill walking; solid red line: CP level walking; dashed red line: CP uphill walking).

This gait adjustment strategy agrees with the results from previous studies [34] using healthy participants, which shows that healthy adults walking on a slope increased hip flexion, knee flexion, and ankle dorsiflexion to increase toe clearance. However, it is noted that, during level treadmill walking, children with CP had a pathological gait pattern with greater knee flexion and ankle dorsiflexion during the stance phase compared with TD children (see Figure 2). Uphill walking requires more knee flexion and ankle dorsiflexion during the stance phase and increased the severity of the pathological gait.

The ankle angle at the *initial contact* (IC) showed a significant group∗walking condition interaction effect. The interaction effect means that slope walking influenced ankle dorsiflexion at the IC more in CP than in TD children and influenced knee extension less in CP than in TD children. The difference may be due to spasticity of muscles, limiting the range of motion in the CP group and the adaptation ability of CP and TD children for the different walking conditions. Besides, uphill walking requires a significant effort to propel the body upwards. Previous research shows that compared with level treadmill walking condition, the peak hip extension moment, peak knee extension moment, and peak ankle plantar flexion moment are significantly higher when walking uphill at the same speed [13]. Our results show that there are no significant differences in peak knee extension moment and peak ankle plantar flexion joint moment for the two walking conditions. This finding may be caused by the slower walking speed for uphill walking, which can be explained as a strategy to reduce joint loading [13].

In the frontal plane, a significant between-group difference is observed for hip abduction moment. This is under expectation as TD children have wider steps, which results in a larger moment arm of the ground reaction forces. We find that the uphill walking also results in greater pelvic oblique angles and decreased hip abduction angles compared to level treadmill walking, which may be a strategy to maintain balance in the *medial-lateral* (ML) direction as these changes will move the COM more close to the COP in the ML direction. In addition, the trunk rotation angle shows a significant group∗walking condition interaction effect. This means that uphill walking influenced trunk rotation more in TD than in CP. Further research is expected to investigate the contributing factors for trunk motion strategies during slope walking.

Compared to level treadmill walking, uphill walking has a significantly less COM-COP distance in the anterior direction. The significant difference may be caused by the smaller inclination angle during uphill walking conditions [35]. No between-group difference is identified for the COP-COM distance in the lateral direction. These results are a bit surprising given that children with CP are reported to have larger displacements of the COP and COM in the medial-lateral direction [29]. This may also be affected by the COM velocity in the ML direction.

To the best of the authors' knowledge, this is the first time a comprehensive 3D kinematics and kinetics as well as the dynamic stability analysis (except for some angles in the transverse planes) performed for CP children during slope walking under a virtual reality environment.

Our findings have some clinical implications. As evident from Figure 2, CP children need to generate extra ankle plantar flexion moment during the early stance phase with a crouched posture (excessive ankle dorsiflexion and knee flexion). This finding agrees with Hösl et al. [16], who observes the increased activation of the calf muscles for CP children during the early stance phase. A biomechanical study shows that the peak knee joint force could be greater than six times the body-weight for severe crouch gait [35]. Crouched gait also could cause joint pain and decrease walking ability [36]. In a study with obese patients, it is shown that uphill walking with a slower speed could reduce the joint loading (peak knee extension and adduction moments) [16]. We suggest that, during a treadmill training with an inclination, the walking speed should be carefully controlled so that peak joint loading will not increase too much. Using a partial weight support system during treadmill training may reduce some joint load for patients.

Studies on single measures of the overall gait pathology such as the *Gait Deviation Index* (GDI) [37], *Gait Profile Score* (GPS), and *Movement Analysis Profile* (MAP) [38] have shown their effectiveness in clinical scenarios. Such outcome measures could assess the overall severity of walking or evaluate the overall performance of an intervention the patient received to improve gait ability. A further study is needed to investigate the overall gait pathology for the CP children during inclined walking under a virtual reality environment using an index like the GPS or MAP.

The study has a small sample size, with ten participants in each group. The CP group also does not distinguish between crouch gaits with apparent equines. These issues affect statistical power to a certain extent. Studies with a larger sample size are required to testify these results and to investigate the relationship between pathological gait patterns, gait functions, GFMCS, spasticity, muscle force, and dynamic balance during inclined walking or other different environments in daily life.

## 5. Conclusion

CP children showed similar adjustments in their gait during uphill treadmill walking under a virtual reality environment as TD children. CP children could maintain similar dynamic balance with a lower walking speed when walking uphill. Uphill walking magnifies the existing abnormal gait patterns of the CP children. During a treadmill training with an inclination, the walking speed should be carefully controlled in the case of improving peak joint loading too much.

## Figures and Tables

**Table 1:** Characteristics of participants.

Patient	Age (year)	Gender	Height (cm)	Weight (kg)	Affected side	GFMCS level	Gait type
S1	7	Male	125	30	L, R	II	Mild crouch
S2	7	Female	114	20	L, R	I	Mild crouch
S3	6	Female	131	27	L, R	I	Crouch
S4	8	Female	125	22.5	L, R	I	Mild crouch
S5	6	Male	117	21	L, R	I	Mild crouch
S6	7	Male	122	22.5	L, R	II	Mild crouch
S7	11	Male	145	37	L, R	II	Apparent equines
S8	10	Male	140	36	L, R	II	Apparent equines
S9	12	Female	146	32	L, R	I	Crouch
S10	11	Male	127	30	L, R	II	Apparent equines

Abbreviations: GMFCS = Gross Motor Function Classification System; L = left; R = right.

**Table 2:** Segment coordination systems.

Segment	Definition of the segment coordination system	
Pelvis	Origin	Midpoint between hip joint centers
	X	Unit vector of cross product between the *Z*-axis and the vector from right hip joint center to left hip joint center
	Y	Unit vector defined by the *X*-axis and *Y*-axis to create a right-hand coordinate system
	Z	Unit vector parallel to the line from S1/L5 to the midpoint between left and right shoulder joint centers
Torso	Origin	Thoracolumbar joint center
	X	Unit vector perpendicular to the plane formed by the *Z*-axis and the vector from right shoulder joint center to left shoulder joint center
	Y	Unit vector defined by the *X*-axis and *Y*-axis to create a right-hand coordinate system
	Z	Unit vector parallel to the line from S1/L5 to the midpoint between left and right shoulder joint centers
Thigh	Origin	Hip joint center
	X	Unit vector perpendicular to the *Z*-axis lies in the global sagittal plane and points anteriorly
	Y	Unit vector defined by the *X*-axis and *Y*-axis to create a right-hand coordinate system
	Z	Unit vector from knee joint center to hip joint center
Shank	Origin	Knee joint center
	X	Unit vector perpendicular to the *Z*-axis lies in the global sagittal plane and points anteriorly
	Y	Unit vector defined by the *X*-axis and *Y*-axis to create a right-hand coordinate system
	Z	Unit vector from ankle joint center to knee joint center
Foot	Origin	Subtalar joint center
	X	Unit vector perpendicular to the *Z*-axis lies in the global sagittal plane and points anteriorly
	Y	Unit vector defined by the *X*-axis and *Y*-axis to create a right-hand coordinate system
	Z	Unit vector from toe joint center to subtalar joint center

**Table 3:** Descriptive statistics for key gait variables of CP and TD children under two walking conditions (level and uphill treadmill walking) and results of two-way ANOVA for differences in the group (CP or TD children), walking condition, and group∗walking condition interaction.

Parameters	Level				Uphill (+10 degree)				*p* value of ANOVA		
	CP		TD		CP		TD		Group	Walking condition	Interaction
	Mean	SD	Mean	SD	Mean	SD	Mean	SD			
Speed (m/s)	0.42	0.16	0.64	0.06	0.32	0.14	0.58	0.07	**<0.01**	**<0.01**	0.494
Stride length (m)	0.52	0.19	0.68	0.12	0.39	0.16	0.65	0.14	**0.003**	**<0.01**	**0.001**
Step width (m)	0.09	0.02	0.12	0.04	0.09	0.03	0.11	0.04	0.05	0.135	0.199
Stance phase (%)	71.12	4.23	66.2	0.92	73.95	3.5	67.49	1.07	**<0.01**	**<0.01**	0.063
Peak trunk flexion (°)	8.12	4.07	6.01	1.85	7.21	4.32	4.56	3.1	0.069	0.228	0.779
Peak trunk extension (°)	-2.7	2.75	-0.16	1.38	1.06	4.48	0.62	3.85	0.375	**0.026**	0.132
Peak trunk rotation (°)	4.84	8.90	4.96	6.67	2.86	8.53	9.21	5.23	0.493	0.224	**0.017**
Peak trunk lateral flexion (°)	-2.30	6.92	6.36	2.50	8.28	6.01	4.50	3.66	0.226	0.241	0.47
Peak pelvic anterior tilt (°)	12.46	5.2	12.93	4.35	26.07	6.94	26.3	7.38	0.88	**<0.01**	0.865
Peak pelvic posterior tilt (°)	7.34	4.49	8.9	4.48	21.01	7.13	21.9	7.7	0.593	**<0.01**	0.682
Peak pelvic oblique (°)	-2.88	7.28	-2.53	3.29	-5.86	7.55	-5.47	4.30	0.95	**<0.01**	0.941
Peak hip flexion (°)	39.81	9.32	38.91	7.33	49.65	11.4	52.5	10.26	0.786	**<0.01**	0.292
Peak hip extension (°)	6.62	7.62	3.36	6.61	11.28	7.26	7.16	8.36	0.182	**<0.01**	0.684
Peak hip abduction (°)	9.98	10.18	9.85	3.77	8.47	9.36	6.33	2.96	0.816	**0.026**	0.28
Peak hip adduction (°)	2.74	16.79	4.62	4.99	1.30	11.20	5.18	5.03	0.581	0.761	0.459
Peak knee flexion during LR (°)	27.15	6.43	20.54	9.95	44.63	6.7	34.66	10.09	**<0.01**	**<0.01**	0.333
Peak knee flexion (°)	60.74	8.11	65.63	11.18	60.58	7.72	67.06	5.44	**0.044**	0.546	0.454
Peak knee extension (°)	12.75	6.9	4.23	4.8	14.61	7.24	10.49	6.57	**<0.01**	**<0.01**	0.063
	Mean	SD	Mean	SD	Mean	SD	Mean	SD			
Peak ankle dorsiflexion (°)	17.55	6.53	11.86	3.59	24.18	5.81	18.64	4.3	**<0.01**	**<0.01**	0.932
Peak ankle plantarflexion (°)	-5.58	7.62	-14.27	6.14	2.73	7.36	-9.57	6.64	**<0.01**	**<0.01**	0.174
Knee flexion at IC (°)	23.49	7.86	6.93	6.01	43.88	6.21	26.74	13.21	**<0.01**	**<0.01**	0.878
Ankle sagittal angle at IC (°)	-1.14	8.18	-5.43	4.6	11.31	7.05	1.46	5.82	**<0.01**	**<0.01**	**0.004**
Peak hip extension moment (N^∗^m/kg)	0.54	0.18	0.36	0.09	0.79	0.19	0.55	0.15	**<0.01**	**<0.01**	0.395
Peak hip flexion moment (N^∗^m/kg)	-0.17	0.07	-0.16	0.07	-0.10	0.05	-0.08	0.04	0.398	**<0.01**	0.852
Peak hip abduction moment (N^∗^m/kg)	0.44	0.21	0.62	0.12	0.39	0.14	0.54	0.09	**0.018**	0.113	0.596
Peak knee abduction moment (N^∗^m/kg)	0.11	0.05	0.10	0.05	0.12	0.06	0.15	0.08	0.898	0.066	0.179
Peak knee adduction moment (N^∗^m/kg)	0.11	0.11	0.12	0.11	0.11	0.13	0.12	0.11	0.737	0.78	0.962
First peak knee extension moment (N^∗^m/kg)	0.14	0.16	0.15	0.08	0.09	0.12	0.23	0.15	**0.032**	0.657	0.057
Peak knee flexion moment (N^∗^m/kg)	-0.24	0.14	-0.22	0.19	-0.24	0.15	-0.25	0.11	0.908	0.423	0.584
First knee peak flexion moment (N^∗^m/kg)	-0.23	0.15	-0.19	0.11	-0.23	0.16	-0.24	0.13	0.82	0.368	0.392
Peak ankle plantarflexion moment (N^∗^m/kg)	0.76	0.26	0.99	0.19	0.74	0.16	0.91	0.21	**<0.01**	0.255	0.545
Peak ankle dorsiflexion moment (N^∗^m/kg)	-0.05	0.06	-0.09	0.05	-0.02	0.01	-0.06	0.03	**<0.01**	**0.01**	0.996
COM-COP anterior distance (m)	0.12	0.05	0.14	0.05	0.03	0.04	0.06	0.05	0.077	**<0.01**	0.838
COM-COP posterior distance (m)	0.09	0.08	0.22	0.19	0.14	0.14	0.27	0.13	0.088	0.092	0.764
COM-COP medial distance (m)	0.15	0.04	0.15	0.02	0.16	0.04	0.14	0.02	0.696	0.628	0.555
COM-COP lateral distance (m)	-0.09	0.04	-0.04	0.04	-0.08	0.07	-0.03	0.03	0.07	0.32	0.624

Abbreviations: LR = load responding; IC = initial contact; CP = cerebral palsy; TD = typically developing.

## Data Availability

The data that support the findings of this study are available on request from the corresponding author, Ye Ma. The data are not publicly available yet due to the underdevelopment of the system and the ethics of the project.
